# Embolized Stems Recover Overnight in *Zea mays*: The Role of Soil Water, Root Pressure, and Nighttime Transpiration

**DOI:** 10.3389/fpls.2017.00662

**Published:** 2017-04-28

**Authors:** Sean M. Gleason, Dustin R. Wiggans, Clayton A. Bliss, Jason S. Young, Mitchell Cooper, Katie R. Willi, Louise H. Comas

**Affiliations:** Water Management and Systems Research Unit, United States Department of Agriculture – Agricultural Research Service, Fort CollinsCO, USA

**Keywords:** xylem, plant hydraulics, embolism, embolism recovery, hydraulic conductivity, micro-CT

## Abstract

It is not currently well-understood how much xylem conductance is lost in maize plants during the day, if conductance is recovered during the night, or what soil water conditions are required for recovery to take place. To answer these questions we designed a greenhouse experiment whereby two genetically dissimilar maize genotypes were subjected to a level of water stress commonly experienced in the field (Ψ_xylem_ ∼-2 MPa). We then measured the loss of stem-specific conductivity associated with this level of stress, as well as the overnight recovery following three re-watering treatments: Ψ_soil_ ∼ 0 MPa, Ψ_soil_ ∼-0.40 MPa, and Ψ_soil_ ∼-1.70 MPa. Mid-day leaf water potentials of -1.98 MPa resulted in stem-specific conductivity (K_S_) values that were 31.5% of maximal (i.e., 68% loss). Returning soils to field capacity (Ψ_soil_ ∼ 0 MPa) overnight allowed for the significant recovery of K_S_ (76% of maximal), whereas partial watering (Ψ_soil_ ∼-0.40 MPa) resulted K_S_ values that were 51.7% of maximal values, whereas not watering resulted in no recovery (35.4% of maximal; Ψ_soil_ ∼-1.7 MPa). Recovery of K_S_ was facilitated by the generation of root pressure and low rates of nighttime transpiration.

## Introduction

Water transport through the xylem tissue represents a physiological linchpin, whereby its loss or meaningful decline results in the direct reduction of CO_2_ conductance and growth. Considering that gas exchange is dependent on the supply of water transferred through the xylem tissue, the supply of this water must be adequate to support maximal stomatal conductance, but also be sufficiently guarded against embolization and dysfunction. Efforts to improve crop species, therefore, depend critically on understanding the processes leading to the loss of hydraulic conductivity, as well as its repair. Previous research has shown that maize loses ca 50% of root and stem-specific hydraulic conductivity daily (McCully, 1999; Li et al., 2009). Similarly, daily/seasonal conductivity loss and recovery has been reported in other angiosperm species (Hacke and Sauter, 1996; Salleo et al., 1996; McCully, 1999; Tyree et al., 1999). Although the conditions and mechanism whereby embolized vessels regain conductivity is presently poorly understood, it is likely to require either full or partial relaxation of hydrostatic tension existing in xylem. This would include refilling under reduced tension (Salleo et al., 1996; Tyree et al., 1999; Zwieniecki and Holbrook, 2009), as well as refilling under neutral or positive pressure, i.e., root pressure (Hacke and Sauter, 1996; Tibbetts and Ewers, 2000; Clearwater et al., 2007; Saha et al., 2009; Cao et al., 2012). Here, we address the ability of maize to recover from conductivity loss, as well as the soil water conditions and xylem water potentials required for this recovery to occur.

In comparison with woody dicotyledons, little is known about water transport and the hydraulic functioning of grasses and other monocotyledon species (Ocheltree et al., 2014, 2016). This is especially surprising considering that grasses are the dominant component in ca 40% of earth’s ecosystems, and contribute more to food and biofuel production than any other life form (Jacobs and Everett, 1998; Maity, 2016; Ocheltree et al., 2016). Little is known about how monocots develop root pressure, how wide-spread it is across taxa, nor if it facilitates vessel refilling. Therefore, research is currently needed to improve hydraulic methods for monocot species, as well as hydraulic methods more generally. For example, it has recently been suggested that estimates of conductivity loss, and therefore also its recovery, have been widely and egregiously overestimated, as a result of a methodological ‘cutting artifact’ (Cochard and Delzon, 2013; Wheeler et al., 2013; Torres-Ruiz et al., 2015). Proponents of this theory have demonstrated that the process of cutting xylem whilst under tension introduces embolism to otherwise water-filled vessels. Others assert that studies reporting the cutting artifact are themselves troubled by artifacts, and that by allowing xylem segments to rehydrate they are observing speedy refilling, rather than the absence of embolism (Trifilo et al., 2014).

Considering that gas exchange and growth depend directly on the functioning of xylem tissue, it is imperative that this issue be resolved, particularly in species of key economic importance. Maize represents, arguably, the most important crop species world-wide, and also an ideal system to determining the conditions necessary for vessel refilling. This is because maize stems are separated discretely into node and internode sections, with bordered pits occurring only within node sections (Shane et al., 2000). As such, relatively short sections can be cut free of the stem with near-absolute certainty that emboli (native or via artifact) cannot pass through the bordered pits present in the nodes. This facilitates conductivity measurements using the standard ‘Sperry apparatus’ (Sperry et al., 1988) after relaxing hydrostatic tension (Wheeler et al., 2013), as well as the direct observation of gas-filled vessels under tension via x-ray micro-computed tomography (μCT) (Cochard et al., 2015) (see Materials and Methods).

We measured the loss of conductivity occurring in greenhouse maize plants during a ‘dry-down’ experiment. Maize plants were allowed to gradually dry down over the course of several days until mid-day leaf water potentials were between -1.6 and -2.2 MPa, representing losses of ca 50–70% of stem conductivity, i.e., a level of loss commonly experienced by even well-watered plants in the field (Tyree et al., 1986). In the evening, plants were subjected to three treatments whereby pots were either fully watered (until freely draining), partially watered (to achieve pre-dawn leaf water potentials of ca -0.40 MPa), or given no water. The following morning, hydraulic functioning was measured using standard techniques and μCT. At the same time, pre-dawn root pressure, and root flow rate (from severed root systems) were also measured. Our study addressed three questions. First, can the degree of stem conductivity that is commonly lost during the day in maize be recovered overnight, and if so, how complete is this recovery? Second, are root pressure and root flow rate aligned with the recovery of conductivity? Third, what soil and xylem water potentials are required for this recovery to take place, and how important are root pressure and nighttime transpiration in facilitating this process?

## Materials and Methods

### Plant Selection and Greenhouse Conditions

Inbreds B73 and CML103 from the maize nested association mapping (NAM) population were used in this study (Yu et al., 2008). We chose genotypes from the NAM population because they are ‘open-source’ and designed to provide a model for evaluating the genetic underpinnings of complex traits, such as drought tolerance (Yu et al., 2008; Gore et al., 2009). We chose inbred B73 because its genome has been completely sequenced, and the data reported here are part of a larger effort to improve the species. We chose CML103 because it had demonstrated enhanced xylem safety (P_50_) in a previous field experiment. Seed was provided by USDA’s North Central Regional Plant Introduction Station, Ames, IA, USA.

Plants were grown in 13.7-L pots filled with Turface Greens Grade soil conditioner (Profile Products, Buffalo Grove, IL, USA) at the USDA – ARS Water Management and Systems Research Laboratory, Fort Collins, CO, USA. Turface is a kiln-fired porous ceramic material with a high infiltration rate. Filled pots were arranged on two greenhouse benches in four lines of 29 pots for a total of 116 pots (58 plants of each genotype). Each individual row consisted of two border plants on each end with 25 randomized experimental plants in-between. All pots were re-randomized twice during the experiment (15 January and 21 January). High pressure sodium lights operated from 0600 to 2000 and supplemented ambient solar radiation by providing an additional 300–600 μmol m^-2^ s^-1^ of PAR. Maximum daily PAR levels ranged from ca 522 to 1464 μmol m^-2^ s^-1^. Mean air temperature at 0900 and 1400 was 27.3°C (*SD* = 2.8; *n* = 105) and 30.7°C (*SD* = 3.6; *n* = 105), whereas mean relative humidity during these same times was 41.9% (*SD* = 12.7; *n* = 105) and 15.3% (*SD* = 7.6; *n* = 105).

Three seeds were planted in each plot on December 14 to a depth of 3.8 cm. Emergence occurred on December 24, and plants were thinned 2 weeks later to include one healthy plant per pot. Pots were watered to field capacity every day at 0500 and 1600 for 4-min (each irrigation providing ca 500 ml of water) via a drip-line irrigation system. The drip-line system consisted of 1.9-cm diameter poly tubing connected to a battery operated timer (RBC7000, DIG, Corp., Vista, CA, USA), with backflow preventer, 207-kPa pressure regulator (Landscape Products, Inc., Tolleson, AZ, USA), and in-line filter (Landscape Products, Inc., Tolleson, AZ, USA) placed between the tubing and the greenhouse irrigation valve. One 7.6-L-h^-1^ emitter (Rainbird, Corp., Tucson, AZ, USA) was attached to the main poly-line using 0.64 cm diameter distribution tubing inserted into each pot with a plastic stake. Fertilizer was applied by top-dressing each pot with UFLEXX 28-3-10 Turf Fertilizer (Howard Johnson’s Enterprises, Inc., Milwaukee, WI, USA) to obtain 71 mg N L^-1^ dry soil on five occasions throughout the experiment. Additional fertilization consisted of each pot receiving general purpose water soluble fertilizer (20-20-20) mixed to obtain 7.6 mg N L^-1^ dry soil four times during the experiment.

### Treatments and Sampling

Plants remained fully watered until they reached V10 (10 leaves present and ca 0.8 m tall), at which time water was withheld from all plants in the experiment. Whole-pot evapotranspiration was monitored (1-g precision) by placing eight plants on digital balances (Adam CBK 70A, Adam Equipment, Inc., Oxford, CT, USA) and recording mass continuously in 1-min time steps via data-logger (constructed in-house using a Raspberry Pi 2, Model B with Rasclock RTC). Four plants of each genotype were placed on these balances and remained on them throughout the experiment. Of the plants placed on balances, two plants of each genotype were randomly assigned to the fully watered and no water treatments. Evaporation from the soil surface was measured during pre-dawn hours by applying the same watering treatments to pots without plants and logging their mass, as above. This was done, rather than wrapping the tops of the pots with plastic, to keep water treatments and rates of loss identical between plants on balances and plants on the bench. Canopy transpiration was calculated as evapotranspiration minus evaporation. Mid-day leaf water potentials were measured periodically (Model 3005, Soil Moisture Equipment, Corp., Santa Barbara, CA, USA) until the target mid-day leaf water potential (ca -1.8 MPa) was reached. Plants were weighed and hand-watered daily (ca 0900) to maintain weights near the mean measured weight at this target mid-day water potential.

Each evening (1900), 18 experimental plants were randomly chosen on the greenhouse benches. Six plants (three from each genotype) were given 4000 ml of water, which resulted in complete return to field capacity and freely draining pots (‘fully watered’ treatment). Six different plants were given between 300 and 700 ml of water, depending on the soil water content of the pots, which was estimated by placing each pot (plus plant) on a balance. Water volume was adjusted in this way to achieve a pre-dawn xylem water potential ranging between -0.2 and -0.5 MPa (‘partially watered’ treatment). The remaining six plants were given no water (‘no water’ treatment). At 0500 h the following morning pre-dawn leaf water potentials were measured on all 18 plants. The first collared leaf from the top of each plant was cut with a sharp pair of scissors and immediately placed in sealable plastic bag. Bagged leaves were kept in a dark ‘cool-box’ until leaf water potential could be measured, generally within 30 min of collection.

### Stem Cutting and Sample Preparation

We utilize the unique xylem anatomy of maize to facilitate avoidance of a cutting artifact (Wheeler et al., 2013) and for the preparation of short stem segments for μCT analysis. If it is assumed that all vessels end at nodes, then the sap arriving at a node must then be transferred across a pit membrane to continue into an adjoining vessel (Shane et al., 2000). We were unable to pass compressed air (80 kPa) through nodes, indicating that all vessels did indeed terminate at nodes in both genotypes and that gas at this pressure was arrested at the pit membranes. However, this does not preclude the possibility that air from native or introduced emboli (e.g., via a cutting artifact) at high tension could pass through pit membranes. Nevertheless, we feel that this unique anatomy of maize likely minimizes the spread of gas between internode sections and therefore facilitates conductivity measurements using the standard ‘Sperry apparatus’ (Sperry et al., 1988) after relaxing hydrostatic tension (Wheeler et al., 2013), as well as the direct observation of gas-filled vessels under tension via x-ray micro-computed tomography (μCT) (Cochard et al., 2015) (see Materials and Methods). We note, however, that our results cannot be extrapolated to species other than maize, as maize was the focus of this case study.

**FIGURE 1:**
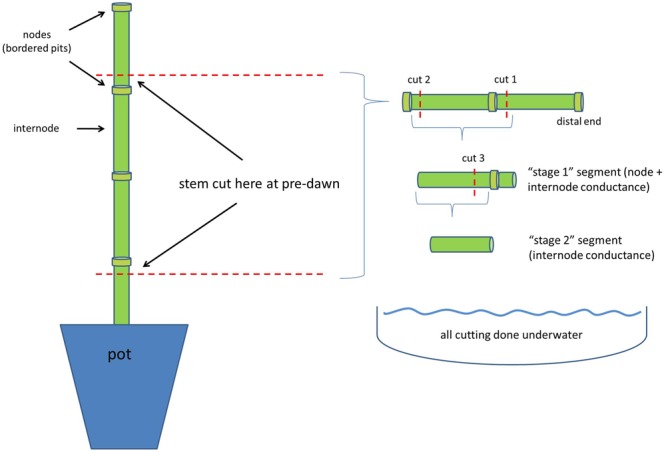
**Cutting diagram for preparing stem segments for measurement.** ‘Call outs’ denote node and internode sections, as well as locations where stems were cut. The right-side portion of the figure shows cutting procedure to rehydrate internode sections. ‘Cut 1,’ ‘cut 2,’ ‘cut 3,’ denote the sequence of stem cutting whilst underwater. The intent of cut 1 was to quickly reduce tension in the internode (Wheeler et al., 2013), but also prevent emboli from entering the internode (gas is blocked by bordered pits present in the node).

Immediately after measuring predawn water potential (0500 h), the aboveground shoots were severed from the root system by cutting each plant near the base (in air), leaving a 2-cm stump remaining in the pot. A single stem section consisting of two internodes and three nodes was cut from the base of each plant, being careful to include nodes at both the top and the bottom of the section (**Figure 1**). Leaves were carefully removed from the nodes and adhesive applied to the cut surfaces (Loctite Liquid, Henkel, Corp., Westlake, OH, USA) to reduce air entry. These prepared stem sections were then sealed inside plastic bags and placed in a dark compartment at room temperature until stem-specific conductivity and μCT scanning could be completed. Stem-specific conductivity was measured on two plants of each treatment each day (for B73 only), whereas one plant of each treatment (both genotypes) was immediately taken to the μCT facility for scanning. This daily procedure was repeated 6 days (22, 23, 24, 25, 26 February, and 2 March), with μCT scanning occurring on February 22 and 23. Water content of the soil was determined gravimetrically for each pot, and converted to soil water potential using a previously published soil water retention curve provided by the manufacturer (van Bavel et al., 1978).

Between 1200 and 1300 h on each of the six measurement days (see above), two plants of each genotype were taken from the greenhouse bench and prepared in an identical fashion as that described above for measurement of stem-specific conductivity during mid-day hours. We interpret this measurement as the lowest stem-specific conductivity value experienced by the plants on that day. Stem-specific conductivity of these samples was measured only on the Sperry apparatus (described below).

### Hydraulic Conductivity, Vessel Refilling

It was found that inbred CML103 did not achieve sufficient stem width for accurate measurement of stem-specific conductivity (stems were too narrow and too soft for measurement). As such, only data from B73 are reported here, which were all of sufficient size. However, μCT analysis of CML103 was still possible and carried out (details below).

Stem-specific conductivity was measured within 4 h of collecting plants (0500–0900 h). Previous research has demonstrated that negligible change in conductance is likely to occur in this time (Alder et al., 1997). However, we also tested this assumption via μCT and also found negligible, albeit a small *increase*, in the percent loss of conductance (PLC) (ca 1.6%) up to 5 h after cutting (Supplementary Figure S2). Stem-specific conductivity was measured on standard ‘Sperry’ apparatus (Sperry et al., 1988). Briefly, stem sections were cut in two stages to allow for the measurement of pit and lumen resistances individually. First, one node and internode were cut free from the section underwater, but the remaining internode (and nodes on either end) were left attached (cut 1 in **Figure 1**). Rapid relaxation of tension was accomplished by allowing the segment to rehydrate for ca 2 min, as suggested by Wheeler et al. (2013), but not significantly more than this (<5 min), to avoid the occurrence of capillary refilling. The remaining nodes (and bordered pits within) kept the internode free of embolism that could possibly have arisen from a cutting artifact. The distal end of this section was then attached to a water-filled silicon tube connected to a four-digit balance. Second, the node on the proximal end of the section was cut off (cut 2, **Figure 1**) and this end was also attached to a water-filled silicon tube, connected to a 2-kPa hydraulic head. Conductance through the section (consisting of one node and one internode) was measured for ca 5 min using filtered (0.2 μm) and deionized water (**Figure 1**). Generally, steady-state conductance was achieved within 10–20 s and was measured via laptop computers interfaced with the balances (three Sperry apparatus were used). After measurement, the stem was again cut (cut 3 in **Figure 1**), this time removing the remaining node and leaving only the internode section, i.e., without bordered pit resistances and protected from cutting artifacts. Conductance was measured again, as previously described, and the internode was then immediately flushed with filtered (0.2 μm) and deionized water for ca 90 s across a 80-kPa pressure gradient. Previous tests have shown that this flushing treatment is sufficient to remove emboli from the now completely open vessels (i.e., all inter-vessel pit resistances were removed with the nodes). After flushing, the internode section was measured again to obtain maximal conductance, i.e., in the absence of gas obstruction and pit resistances. Conductance was normalized by length, cross-sectional area, and corrected for viscosity (20°C); giving stem-specific conductivity. Percent loss of conductivity, from maximal conductivity measured in the fully watered treatment, was calculated for complete stem sections (node and internode sections) as well as for only the internode sections, i.e., without bordered pit resistances.

### X-ray Micro-Computed Tomography (μCT)

Whilst stem-specific conductivity was being measured, the six previously prepared stems (three stems of each genotype) were taken to the μCT facility (the Soft Tissue Mechanics Laboratory, Colorado State University, Fort Collins, CO, USA) and scanned at ca 18-μm pixel^-1^ resolution through the internode sections (Scanco μCT80, Scanco Medical AG, Bruttisellen, Switzerland). Scanning began within 2 h of collection, with the last stem being scanned within 5 h of collection. To ensure no change in PLC had occurred, the first stem scanned was always scanned twice – at the start and at the end of each scanning session (Supplementary Figure S2). Because stem segments were scanned through the internodes, they can only verify the loss of conductance occurring within internodes. Embolized vessels were clearly identifiable in the images, whereas water-filled vessels were indistinguishable from the hydrated tissues surrounding them. All μCT images have been provided in the supplemental materials (Supplementary Figure S1). The total number of meta-xylem vessels in each scanned internode was determined from stained (0.01% safranin-o, Fisher Scientific, Nazareth, NJ, USA) and photographed sections taken on the same internodes using a Nikon SMZ-U dissecting microscope (Nikon, Tokyo, Japan). The fraction of total conductance lost was assumed equal to the number of gas-filled meta-xylem vessels (from μCT), relative to the total number of meta-xylem vessels (from photographs). We note that this procedure will underestimate PLC if wider vessels embolize sooner than narrower vessels. Thus, we use μCT data to verify trends in PLC, rather than magnitudes, i.e., to verify increase in PLC as treatments ranged from fully watered, to partially watered, to no water.

### Root Pressure and Root Flow

Immediately after the plants were cut from the pots, a pre-weighted cotton ball was placed on the cut surface of the stem and a plastic bag was secured over the top to arrest evaporation. Cotton balls were removed after 15 min and re-weighed to calculate the quantity of sap pushed out of the root system through the cut stem (‘root flow rate’) (Zhang et al., 1995). Directly after measuring root flow rate, a 0–0.21 MPa pressure transducer (model PX26-030DV, Omega Engineering, Inc., Stamford, CT, USA) was firmly attached via nested sections (ca 2 cm) of polypropylene tubing (Sperry, 1983). Root pressure was then recorded for 24–48 h via a data-logger (CR1000, Campbell Scientific, Inc., Logan, UT, USA) and the maximum pressure documented during this time was used for analyses.

We calculated the pre-dawn root flow rate that is equal to the pre-dawn transpiration demand in the fully watered treatments for each genotype. We interpret this ‘equalizing’ water potential and flow rate as the *minimum limit* where xylem water potential at the base of the plant could approach zero, and thereby facilitate refilling via positive pressure. We note that our measured root flow rates likely overestimate the flow rates in intact shoots because they do not include resistances in stems and leaves (stems and leaves were cut off to facilitate measurement). As such, the root flow rate that equalizes transpiration represents a conservative limit for when equalizing flow could possibly occur, whereas the water potential corresponding to the equalizing flow in an intact shoot would likely be even closer to zero.

### Statistical Analysis

Differences in measured traits (e.g., PLC, root pressure, transpiration) among treatments and genotypes were compared using ANOVA. Exponential decay (PLC recovery ∼ Ψ_PD_) and logistic (K_S_ ∼ root flow) models were fit using the ‘nlsLM’ function in the minpack.lm package developed for R (Elzhov et al., 2015; R Core Team, 2015). All data were transformed as necessary to meet the assumptions of the analyses.

## Results

### Is Stem-Specific Conductivity Recovered Overnight, and If So, How Complete Is This Recovery?

**FIGURE 2:**
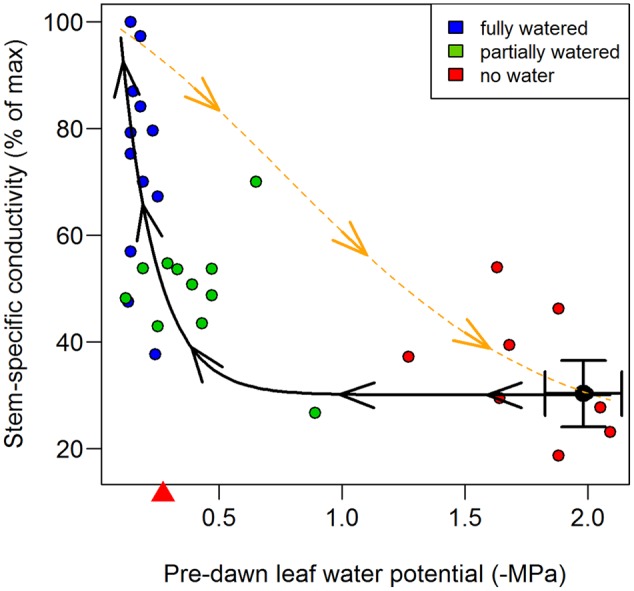
**Stem-specific conductivity loss (orange broken line) and recovery (black solid line) of inbred B73 during water stress.** Recovery was measured at pre-dawn (0500 h) after 10 h of darkness and was significant in both the ‘fully watered’ treatment (blue circles) and in ‘partially watered’ treatments (green circles), but not in the ‘no water’ treatment (red circles). Mid-day stem-specific conductivity and leaf-water potentials are represented by the black circle and error bars (1 SD). Stem-specific conductivity loss data, denoted by the orange broken line (individual points not shown for clarity), were taken from Gleason et al. (2016) and fit with a weibull model. Recovery of stem-specific conductivity was best approximated by an exponential decay model (black solid line) and is shown fit through all observations (red, blue, green symbols). The mean leaf water potential at which flow from the root system (root ‘pressure’) equaled water loss from the canopy in the fully watered treatment is denoted with a red triangle on the x axis, and should be interpreted as the *minimum* water potential at which neutral xylem tension could be expected. Data points represent individual plants.

Mean maximal stem-specific conductivity of internode segments for inbred B73 was 2.12 kg m^-1^ s^-1^ MPa^-1^ (*SD* = 0.20, *n* = 12). At mid-day, stem-specific conductivity had fallen to 31.5% of this value (0.67 kg m^-1^ s^-1^ MPa^-1^; *SD* = 0.20; *n* = 12), corresponding to a leaf water potential of -1.98 MPa (*SD* = 0.31) (**Tables 1, 2**). These results are in close agreement with previously developed PLC curves for maize stems (Li et al., 2009; Gleason et al., 2016). Partial recovery of stem-specific conductivity was observed overnight. Fully watering plants to the point of soil saturation (freely draining pots; Ψ_soil_ ∼ 0), increased stem-specific conductivity to 76.4% (*SD* = 19.2, *n* = 38) of maximal values after 10 h of darkness (Ψ_leaf_ = -0.17 MPa; *n* = 15), indicating significant but not complete recovery from values recorded at mid-day (76.4% vs. 31.5%; *P* < 0.001). In contrast, plants receiving no water overnight exhibited internode stem-specific conductivities not differing from that recorded at mid-day, even after 10 h of darkness (35.4% vs. 31.5%; *P* = 0.958), and had a mean leaf water potential at pre-dawn of -1.71 MPa (*SD* = 0.20; *n* = 12). Intermediate of these two extremes, plants receiving partial re-watering recovered significantly from their mid-day state (51.7% vs. 31.5%; *P* = 0.049), but also fell significantly short of achieving the same recovery as the fully watered plants (*P* = 0.002), and exhibited a pre-dawn leaf water potential of -0.40 MPa (*SD* = 0.20; *n* = 14) (**Table 1**). Taken together, the recovery of internode stem-specific conductivity was associated with pre-dawn leaf water potential and was well-approximated by an exponential decay model (**Figure 2**).

**Table 1:** Effect of watering treatments on stem-specific conductivity (K_S_) measured on the Sperry apparatus and the percent functioning vessels measured via μCT (% functioning) after 10 h of darkness.

Hydraulic functioning	No water	Partially watered	Fully watered	Mid-day
**Sperry apparatus (B73 only)**				
K_S_ internode (% of max)	35.4 (11.7)^bc∗^	51.7 (10.9)^b∗^	76.4 (19.6)^a^	31.5 (12.9)^c^
K_S_ internode (kg m^-1^ s^-1^ MPa^-1^)	0.75 (0.25)^bc∗^	1.09 (0.23)^b∗^	1.62 (0.42)^a^	0.67 (0.27)^c^
K_S_ complete stem (kg m^-1^ s^-1^ MPa^-1^)	0.064 (0.108)^a∗^	0.26 (0.23)^a^	0.29 (0.29)^a∗^	0.082 (0.118)^a^
**μCT scanning**				
μCT, B73 (% functioning)	72.8 (8.7)^a^	72.3 (4.9)^a^	90.1 (9.9)^a^	–
μCT, CML103 (% functioning)	77.7 (4.1)^b^	89.3 (0.3)^a^	95.8 (0.6)^a^	–
μCT, combined (% functioning)	75.2 (6.3)^b^	80.8 (10.2)^ab∗^	92.9 (6.6)^a∗^	–

*‘Mid-day’ values were taken between the hours of 1200 and 1400 immediately after measuring and were not allowed a recovery period. Tukey mean separation (α = 0.05) is denoted with lowercase letters. Borderline significance (α = 0.10) is denoted with an ‘^∗^’. Values in parentheses denote 1 SD. μCT results differed significantly between genotypes (*P* = 0.032).*

**Table 2:** Effect of water treatments on plant water flux and soil and leaf water potentials measured after 10 h of darkness.

Genotypes and traits	No water	Partially watered	Fully watered
**Inbred B73**			
Root pressure (Ψ_R_) (MPa)	0.0005 (0.0005)^b^	0.015 (0.020)^b^	0.078 (0.050)^a^
Root flow rate (Q_R_) (mg s^-1^)	0.009 (0.005)^b^	0.054 (0.070)^b^	0.284 (0.081)^a^
Nighttime evapotranspiration (mg s^-1^)	0.376 (0.134)^b^	–	0.905 (0.219)^a^
Nighttime transpiration (mg s^-1^)	–	–	0.131 (0.146)
Pre-dawn Ψ_leaf_ (MPa)	1.71 (0.28)^a^	0.395 (0.204)^b^	0.173 (0.044)^c^
Pre-dawn Ψ_soil_ (MPa)	0.777 (0.571)^a^	0.274 (0.234)^b^	0.008 (0.016)^b^
**Inbred CML103**			
Root pressure (Ψ_R_) (MPa)	0.0008 (0.0005)^b^	0.014 (0.017)^ab∗^	0.052 (0.047)^a∗^
Root flow rate (Q_R_) (mg s^-1^)	0.009 (0.007)^c^	0.115 (0.132)^b^	0.443 (0.092)^a^
Nighttime evapotranspiration (mg s^-1^)	0.693 (0.240)^b^	–	1.04 (0.204)^a^
Nighttime transpiration (mg s^-1^)	–	–	0.182 (0.106)
Pre-dawn Ψ_leaf_ (MPa)	1.09 (0.36)^a^	0.232 (0.084)^b^	0.138 (0.077)^b^
Pre-dawn Ψ_soil_ (MPa)	0.468 (0.235)^a^	0.190 (0.117)^b^	0.006 (0.009)^c^

*Tukey mean separation (α = 0.05) is denoted with lowercase letters. Borderline significance (α = 0.10) is denoted with an ‘^∗^’. Values in parentheses denote 1 SD.*

**FIGURE 3:**
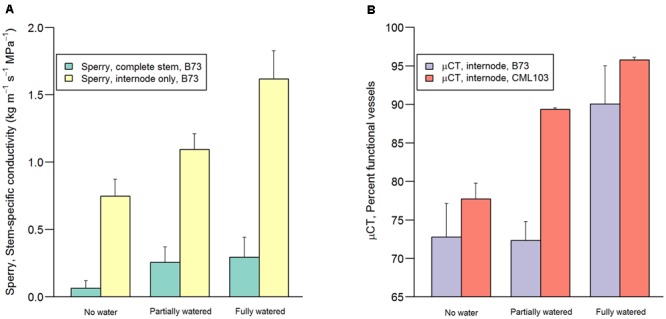
**Stem-specific conductivity measured on the Sperry apparatus (A)** and percent water-filled (functioning) vessels measured via μCT **(B)** in the three water treatments (‘no water,’ ‘partially watered,’ ‘fully watered’) after the overnight recovery period (10 h). Green and yellow bars represent stem-specific conductivity measured in complete stem sections (lumen + pit resistance) and in internodes (lumen resistance) of inbred B73 (left-side y axis). Purple and orange bars represent the percent functional vessels measured in inbreds B73 and CML103 via μCT. Error bars represent 1 SD.

Conductivity of stem sections (internodes + nodes) and internode sections (without nodes) were qualitatively similar in their response to treatments (**Figure 3A** and **Table 1**). However, internode conductivity was ca 5.5 times greater than complete stem conductivity, presumably resulting from resistance contributed by the inter-vessel pits and pit membranes, which were present only in the nodes (Shane et al., 2000) (**Figure 3A**). Markedly greater within-treatment variation was also observed among complete stem measurements compared to internode measurements (**Table 1**). Results from the Sperry apparatus were qualitatively similar to the μCT results, with both methods revealing significant decline in hydraulic functioning with decreasing pre-dawn leaf water potential (**Figure 3B** and **Table 1**).

B73 and CML103 differed in their response to the re-watering treatments, as measured via μCT (*P* = 0.032). CML103 plants in the ‘no water’ treatment exhibited significantly lower percent of functional vessels than plants in ‘fully watered’ treatment (*P* = 0.010) as well as plants in the ‘partially watered’ treatment (*P* = 0.034), whereas B73 plants did not differ significantly in percent functional vessels among any of the re-watering treatments (*P* > 0.221) (**Figure 3B** and **Table 1**). Combining both genotypes in analysis of variance suggested that plants in the ‘fully watered’ treatment had lower percent functional vessels than plants in the ‘no water’ treatment (*P* = 0.009), as well as plants in the ‘partially watered’ treatment (*P* = 0.058), but no difference was evident between ‘no watered’ and ‘partially watered’ treatments (*P* = 0.445) (**Figure 3B** and **Table 1**). We note that the percent loss of functional vessels, as quantified by μCT, should be *less* than the % loss of stem-specific conductivity measured via Sperry apparatus, as we report in **Figure 3** and **Table 1** (Cochard et al., 2015). This result arises for two reasons. Firstly, wider conduits tend to embolize at higher water potential than more narrow conduits (Villar-Salvador et al., 1997; Shane and McCully, 1999; Montwé et al., 2014; Brodribb et al., 2016), yet conductance scales to the 4th power of vessel diameter. Thus, the embolization of the largest vessels, during the early stages of desiccation, results in a proportionately greater loss of stem-specific conductivity than does losing the same number of thinner vessels at latter stages of desiccation. Secondly, μCT and Sperry measurements are themselves quite different methods, which are likely to result in disparate outcomes (Cochard et al., 2015). For example, conductance loss calculated via Sperry apparatus normalizes measurements by a maximal flushed measurement, whereas the μCT method used here normalizes measurements by the total number of vessels in cross-section.

### Are Root Pressure and Root Flow Rate Aligned with the Recovery of Conductivity?

**FIGURE 4:**
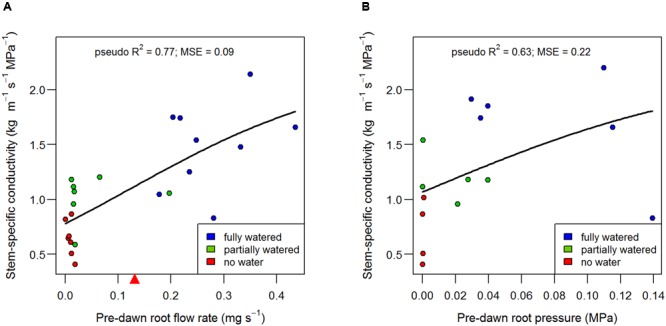
**Alignment of root flow (A)** and root pressure **(B)** with the recovery of stem-specific conductivity measured in B73 internodes. The point where root flow rate equals the rate of transpiration in the fully watered treatments (the point where xylem water potential approaches zero) is denoted by a red triangle on the x axis in **(A)**, and should be interpreted as the *minimum* root flow rate at which neutral xylem tension could be expected. Data points represent individual plants and have been fit with logistic models.

Root pressure and root flow rate (i.e., the rate of sap flowing out of the severed root system) were aligned with stem-specific conductivity (**Figure 4**) and increased in magnitude from the ‘no water’ treatment to the ‘fully watered’ treatment (**Table 2**). The recovery function can be assumed ‘saturating,’ i.e., stem-specific conductivity can approach maximal rates, but not exceed them. As such, the data were fit with a logistic model, which allows for slow (or near-absent) recovery at low flow rates and low pressures, but then approaches maximal stem-specific conductivity at higher flows and pressures (**Figure 4**). Pre-dawn root flow rate explained ca 77% of the variation in stem-specific conductivity (*MSE* = 0.09; *n* = 23), whereas root pressure explained ca 63% of the variation in stem-specific conductivity (*MSE* = 0.20; *n* = 23) when fit with logistic models (**Figure 4**). It is apparent that the ‘best-fit’ logistic model (i.e., that which minimizes residual error on the y axis) may be inappropriate in cases where recovery of conductivity requires a minimum root pressure and flow rate. For example, it has been suggested that conduit refilling cannot take place when xylem is under tension (Cochard and Delzon, 2013), and thus, we might expect recovery of conductivity to occur only when the flow rate from the roots is equal or greater than the rate of water loss from the canopy. Only in such a case can the tension in the xylem be eliminated or reversed, i.e., the generation of positive pressure. Once the transpiration demand is met, then root pressure can ‘push’ water as high as the limit imposed by gravity (Cao et al., 2012).

### What Soil and Xylem Water Potentials Are Required for Recovery to Take Place, and How Important Are Root Pressure and Nighttime Transpiration in Facilitating This Process?

**FIGURE 5:**
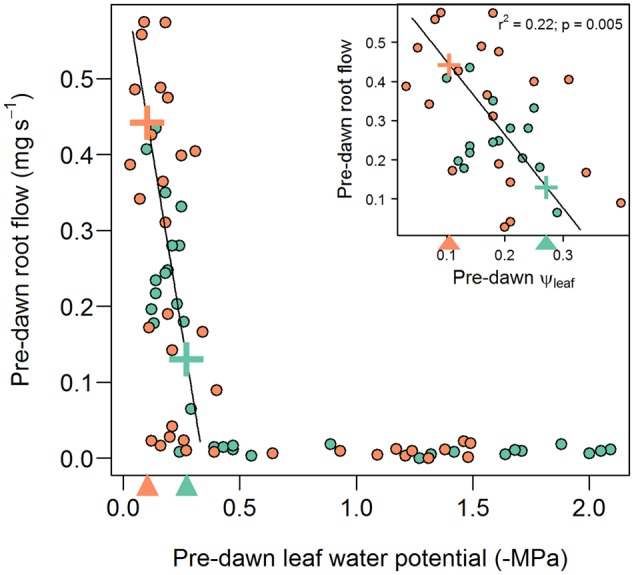
**Relationship between pre-dawn root flow and leaf water potential for inbred B73 (blue circles) and CML103 (orange circles).** Root flow (and therefore also root pressure) does not increase above zero until tension in leaves fall to ca –0.34 MPa, corresponding to a soil water potential of ca 0 MPa. Thereafter (at Ψ_leaf_ > –0.34 MPa), root flow increases linearly with increasing leaf water potential. The insert plot shows only this linear portion of the function. The root flow that balances canopy transpiration in B73 (ca 0.131 mg s^-1^) and CML103 (ca 0.443 mg s^-1^) are denoted with ‘+’ symbols on the fitted line and with triangles on the x axis, and should be interpreted as the *minimum* water potential at which neutral xylem tension could be expected.

Percent loss of conductivity curves have been previously measured on field-grown B73 plants (Gleason et al., 2016) and the fitted Weibull model has been added to **Figure 2** to illustrate the markedly different trajectory of PLC loss (broken orange line) verses recovery (solid black line). Loss of conductance follows the standard inverse sigmoidal shape, whereas recovery (solid black line) was most closely approximated by an exponential decay model, exhibiting significant recovery only when pre-dawn leaf water potentials increased to ca -0.3 MPa (**Figure 2**), corresponding to a soil water potential of between 0 and -0.3 MPa. Thus, it appears that root pressure, and subsequent embolism refilling, is absent unless the soil water potential is at zero or very near zero (**Figure 5**). This result rules out any meaningful chance of embolism refilling during the day when plants are actively transporting water, as well as during the night, when soil water potentials are below ca -0.3 MPa (note the pre-dawn Ψ_leaf_ and root pressure/flow values in **Table 2**).

Pre-dawn nighttime transpiration estimates for B73 and CML103 in the fully watered treatment were 0.131 mg s^-1^ (*SD* = 0.146) and 0.182 mg s^-1^ (*SD* = 0.106), respectively. Pre-dawn root flow rates were about twice this amount, being 0.284 mg s^-1^ (*SD* = 0.081) for B73 and 0.443 mg s^-1^ (*SD* = 0.092) for CML103, suggesting that water potentials at the base of the plants during the night were very near zero. This further suggests that refilling of embolized vessels in maize occurs at xylem water potentials higher than -0.3 MPa, and with little likelihood of refilling at xylem water potentials lower than this. In the fully watered treatment, pre-dawn leaf water potentials ranged from -0.14 MPa in CML103 to -0.17 MPa in B73 (**Table 2**), suggesting that even in the terminal tissues, water potentials were close to zero, and could very well have been zero considering the magnitude of measurement error. More specifically, the 95% confidence interval for the fully watered pre-dawn leaf water potential measurements was 0.13 MPa, suggesting lack of confidence in any pre-dawn measurements higher than ca -0.13 MPa, which is close to the pre-dawn leaf water potential in both genotypes.

## Discussion

Our experiment demonstrates, not only that maize loses a significant fraction of stem-specific conductivity daily, but that conductance can be regained overnight, provided that sufficient water resources are available to the root system. We show that the magnitude of conductivity recovery is correlated, and likely physiologically linked, to the generation of positive root pressure and the subsequent flow of water from the root system. The interaction of soil water potential, canopy transpiration rate, and root pressure result in xylem water potentials at or near zero, resulting in near-complete recovery of stem-specific conductivity during the night. Furthermore, we show that the relationship between the recovery of stem-specific conductivity and leaf water potential exhibits marked non-linearity such that no recovery is evident until soil water and leaf water potentials increase to values close to zero, at which point, recovery increases precipitously. We suggest that these results are notably robust considering that we not only allowed for re-hydration prior to measurement via standard techniques, but also confirmed the loss and recovery of stem-specific conductivity from μCT scans, which allowed for assessment of xylem embolization whilst the stems were under tension.

This has important implications for the management of maize in both dryland (‘rain-fed’) and under limited irrigation. Firstly, it is apparent that growth will be markedly reduced or cease when soil water potential decreases beyond ca -0.3 MPa because routine embolization that occurs during the day, and therefore whole-plant conductance and CO_2_ assimilation, will not recover overnight. As such, plants must be able to access deeper water at night (e.g., hydraulic redistribution), or adequate water must be supplied directly to the upper rhizosphere to enable the recovery of maximal conductance. However, we should not expect meaningful growth and reproductive output at sustained soil water potentials below -0.3 MPa. Furthermore, providing water can be moved up from deeper layers at night, or that water can be applied directly to the surface, complete or near-complete recovery of conductance can be expected, at least in stem xylem. We note that a recent simulation study suggests that rainfall events providing sufficient water to allow for refilling, but insufficient quantities to support transpiration, could lead to even greater rates of xylem desiccation than would occur in the absence of refilling (Sperry and Love, 2015).

Although no other studies have examined xylem embolization, root pressure, and the recovery of conductivity in combination as we have done here, the results of previous efforts do support our main findings. Significant loss of root and stem conductivity during the day in maize has been reported from cryo-scanning electron microscopy experiments (McCully, 1999; Shane and McCully, 1999). Similarly, these studies report recovery of xylem conductivity during the night, however; in contrast to our findings, these studies also report recovery during the day under appreciable rates of transpiration, and presumably, xylem tension (McCully, 1999; Shane and McCully, 1999). Remarkably similar diurnal patterns of petiole conductance (including recovery during daylight hours) have been reported in *Vitis* (Zufferey et al., 2011), a species also known to generate root pressure (Sperry et al., 1987; Tibbetts and Ewers, 2000).

Conduit refilling under tension remains a controversial topic despite significant data in support of it; mostly from studies of woody dicotyledon and gymnosperm species (Nardini et al., 2011; Trifilò et al., 2015; Earles et al., 2016). In contrast to these results, conduit refilling under tension was not observed in *Sequoia sempervirens* saplings via μCT scanning (Choat et al., 2015), nor across a range of Australian conifer species subjected to severe stress (Ψ_leaf_ < P_50_) (Brodribb and Cochard, 2009; Brodribb et al., 2010). Similarly, conductivity loss was not observed in *Laurus nobilis* across a range of xylem tensions that have previously been reported to cause conductivity loss, and thus suggestive of a cutting artifact (Cochard, 2002). It has also been proposed that the refilling controversy is driven mainly by differences between species exhibiting iso- vs. aniso-hydric stomatal response, and as such, we should not expect a common refilling strategy to emerge across vascular species (McCulloh and Meinzer, 2015; Trifilò et al., 2015). We suggest that these conflicting results represent not a pardox, but rather an opportunity. Considering that it is unlikely that plants would exhibit conduit refilling in one study and not another, particularly within the same species, we can likely conclude from this that there are methodological artifacts, as noted by others (Sperry et al., 2012; Cochard et al., 2013; Torres-Ruiz et al., 2014; Trifilo et al., 2014; Hacke et al., 2015). However, even if we subset the body of evidence to that including only direct observations (e.g., μCT, x-ray microscopy, nuclear magnetic resonance), we feel there remains considerable support for the routine loss of xylem conductivity, as well as its recovery in some species (e.g., aniso-hydric), perhaps even under tension (Holbrook et al., 2001; Scheenen et al., 2007; Lee and Kim, 2008; Brodersen et al., 2010; Suuronen et al., 2013; Earles et al., 2016).

## Conclusion

More detailed knowledge of the embolization and refilling processes may not be necessary to improve species via breeding techniques, providing that heritable variation in the underlying traits (e.g., safety, efficiency, recovery) can be measured accurately across genotypes. However, efforts to directly edit the genes themselves will require a more detailed understanding of the physiology and genetic code. As such, we recommend that future research focus on clarifying the proximate causes of embolization (cavitation, emboli expansion, interconduit spread) and recovery (mechanisms, cost, timing) as well as their dependent/coordinated processes (root pressure, nighttime transpiration, soil and atmospheric aridity, carbohydrate reserves). Although maize might serve as an adequate model system, information gained from this system will be of little value if we cannot make broader, and therefore more important, generalizations across species. We suggest that phylogenetically broad surveys of root pressure, nighttime transpiration, and refilling are also desperately needed.

## Author Contributions

SG, DW, CB, MC, and LC: designed the experiment. All authors carried out experiments and assisted with writing the manuscript.

## Conflict of Interest Statement

The authors declare that the research was conducted in the absence of any commercial or financial relationships that could be construed as a potential conflict of interest.
