# Supplementary material for: Embolized Stems Recover Overnight in Zea mays: The Role of Soil Water, Root Pressure, and Nighttime Transpiration
Source: Front Plant Sci. 2017 Apr 28;8:662. doi: 10.3389/fpls.2017.00662 (PMC5408072; doi:10.3389/fpls.2017.00662)
Supplement: Supplementary file 1 [file Data_Sheet_1.DOCX]

Sean M. Gleason*, Dustin R. Wiggans, Clayton A. Bliss, Jason S. Young, Mitchell Cooper, Katie R. Willi, Louise H. Comas

* Corresponding author. Tel.: +1 9704927411

sean.gleason55@gmail.com

**Supplemental Figure S1.** Micro-CT images of stem internodes. The large black sections in the center of most images are non-functional pith and were not included in the analyses. Embolized vessels are seen as small black dots (two examples are circled in red), whereas functional vessels are not visible because the density of water and the surrounding matrix are too similar to provide contrast). The total number of vessels in each internode segment were determined from corresponding light-microscope images (not shown).

Fully-watered


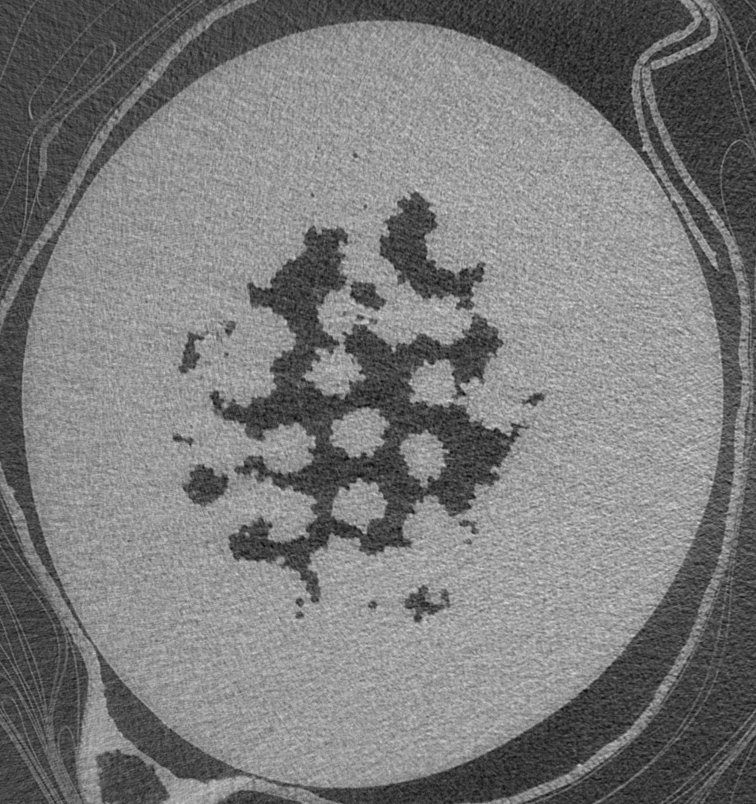

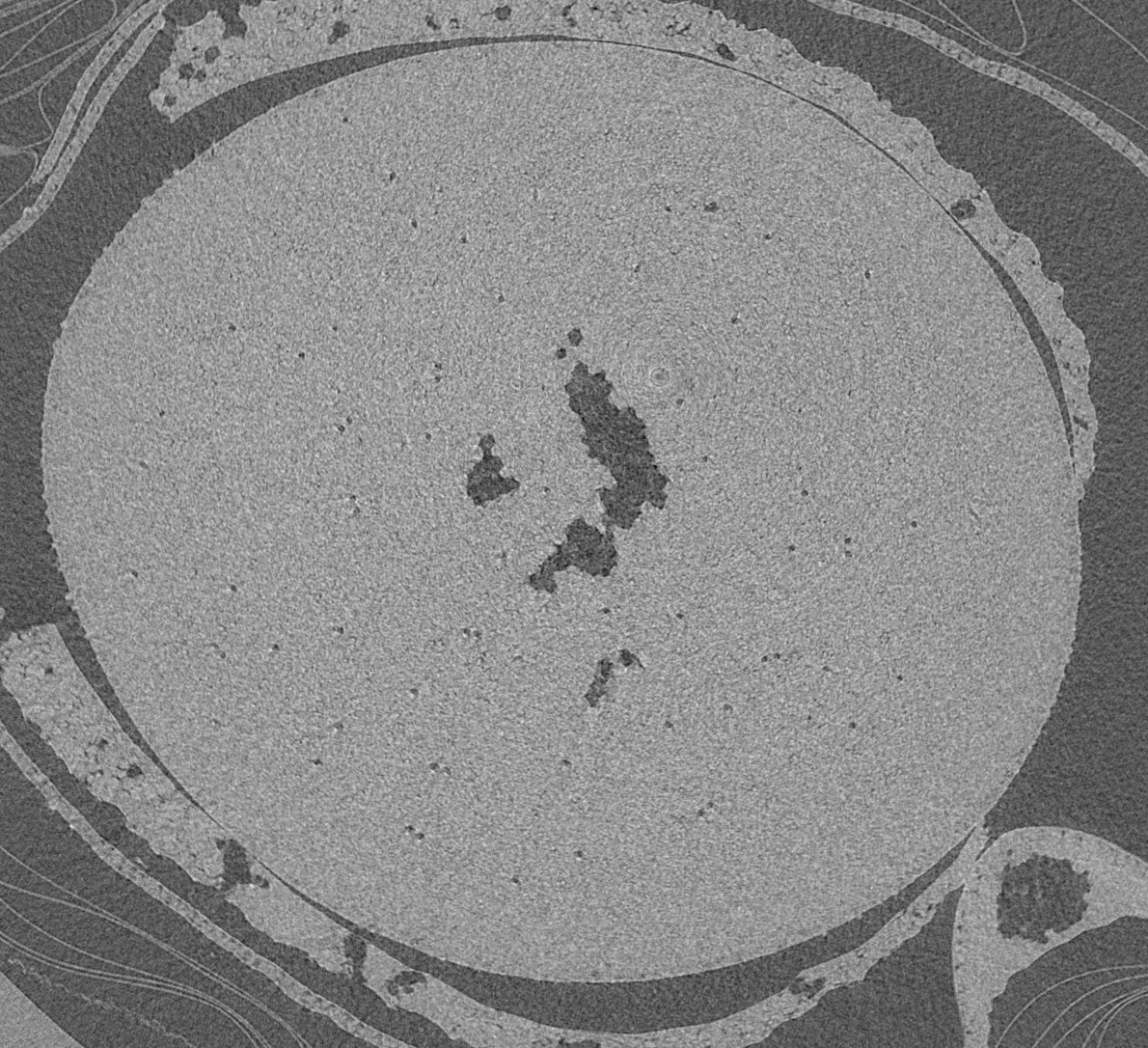


B73, fully-watered, Ψ_PD_ = -0.10MPa, Ψ_MD_ = -1.8

B73, fully-watered, Ψ_PD_ = -0.18MPa, Ψ_MD_ = -1.7


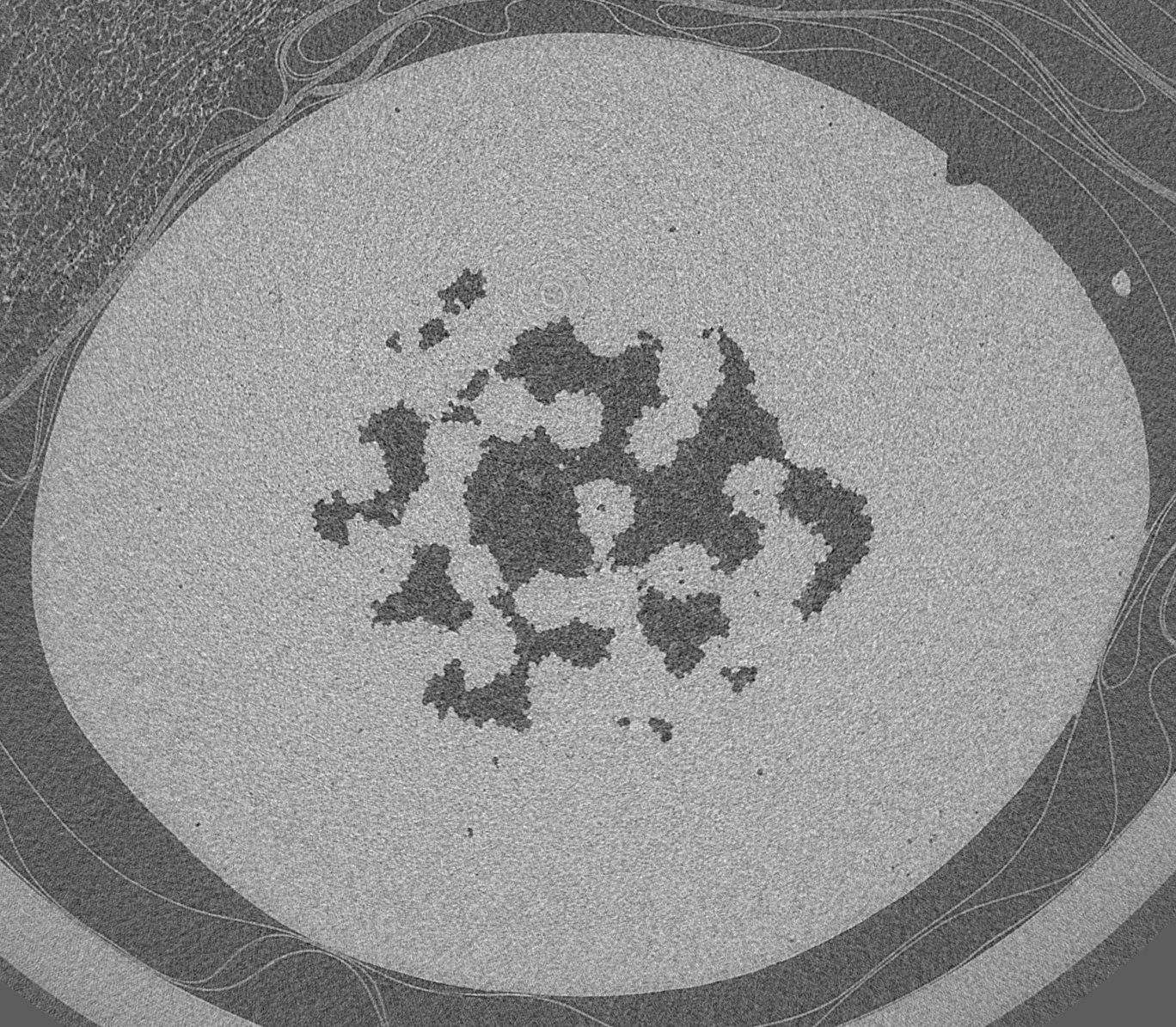

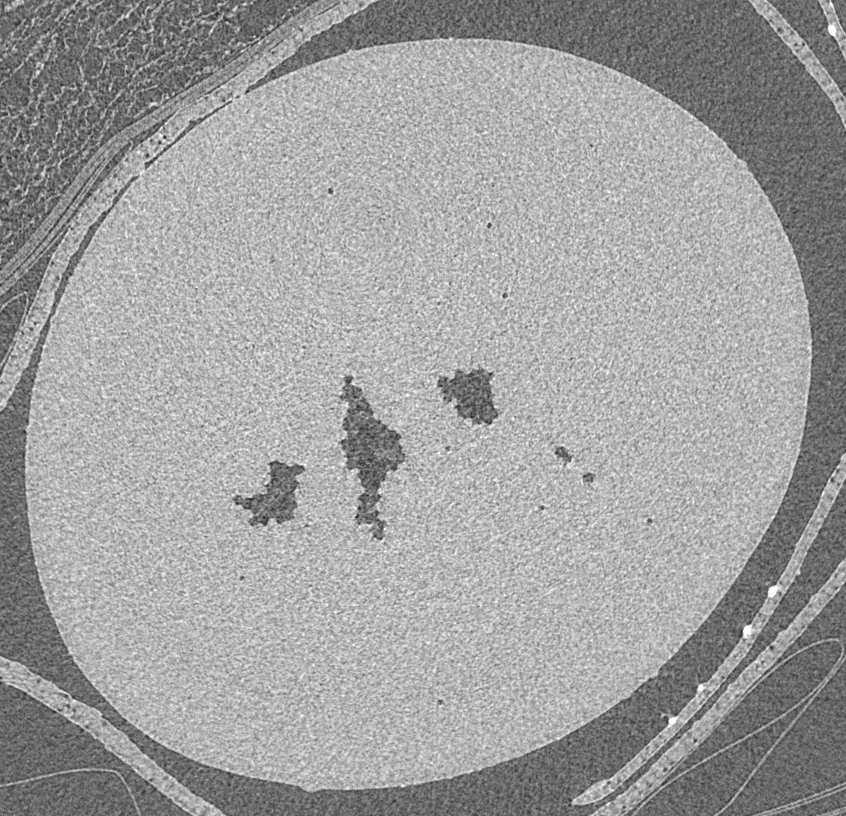


CML103, fully-watered, Ψ_PD_ = -0.18MPa, Ψ_MD_ = -1.5

CML103, fully-watered, Ψ_PD_ = -0.18MPa, Ψ_MD_ = -1.4

Partially watered


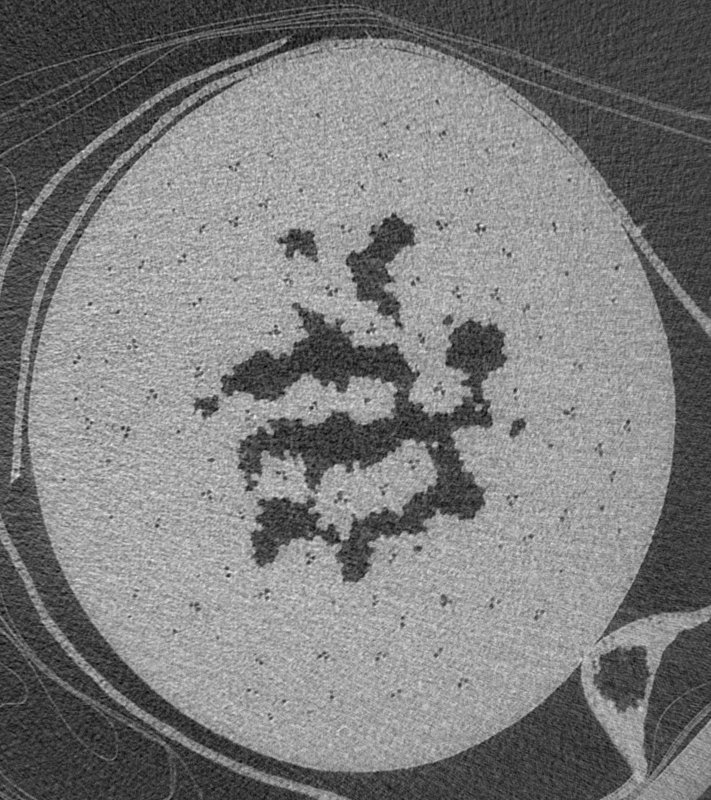

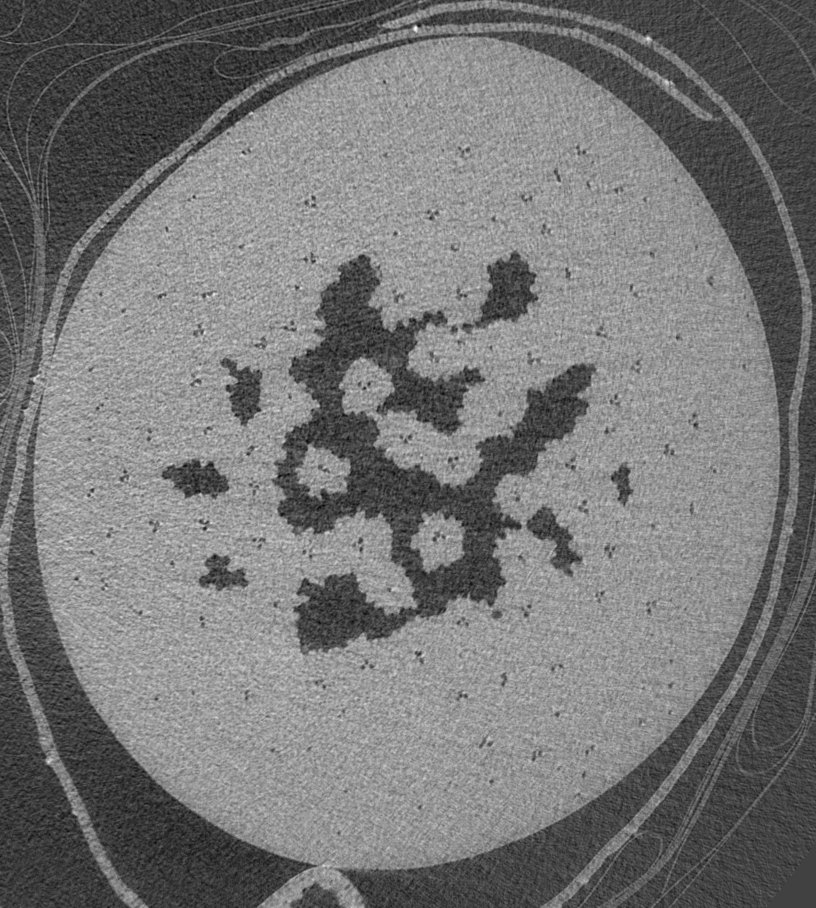


B73, partially-watered, Ψ_PD_ = -0.55MPa, Ψ_MD_ = -1.7

B73, partially-watered, Ψ_PD_ = -0.26MPa, Ψ_MD_ = -2.0


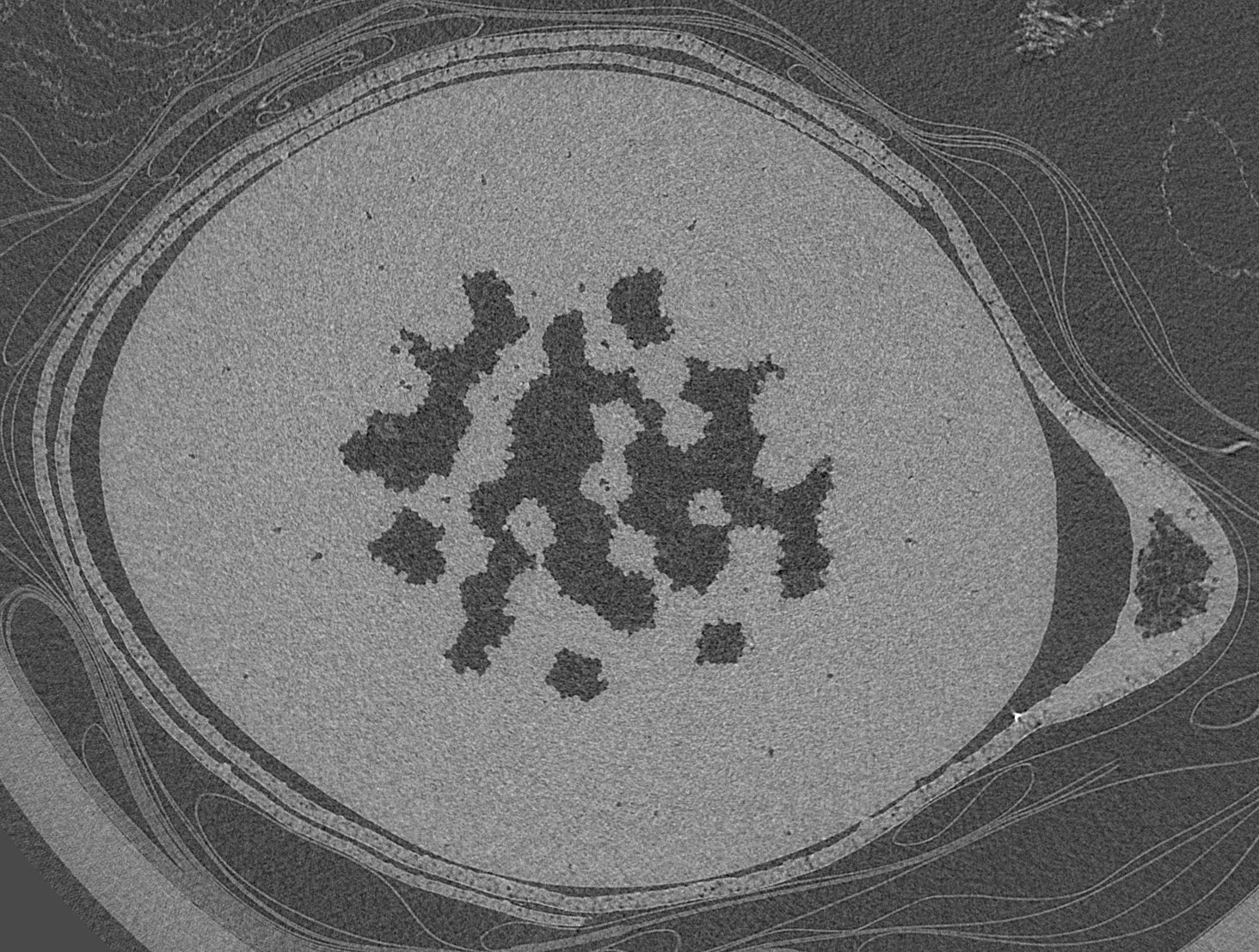

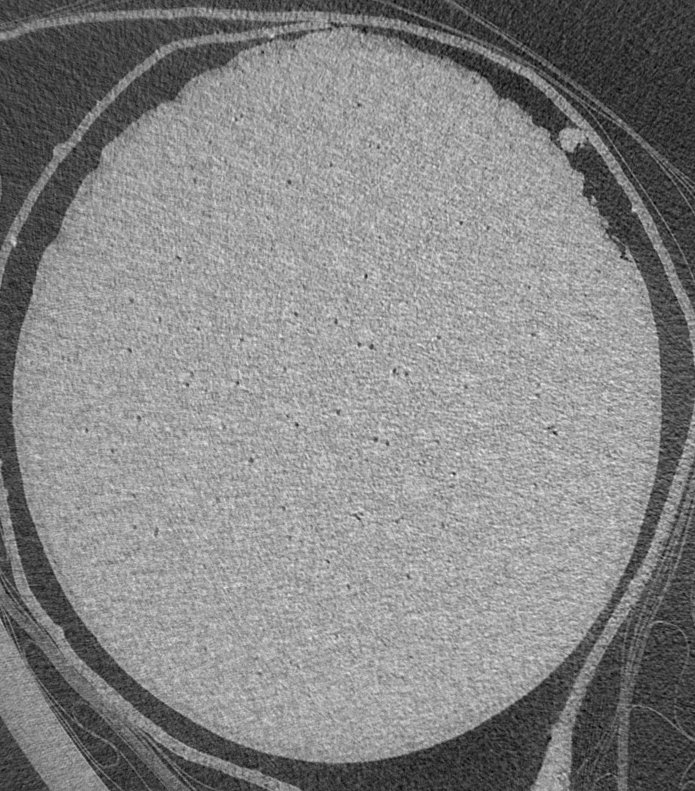


CML103, partially-watered, Ψ_PD_ = -0.19MPa, Ψ_MD_ = -1.5

CML103, partially-watered, Ψ_PD_ = -0.27MPa, Ψ_MD_ = -1.4

No water


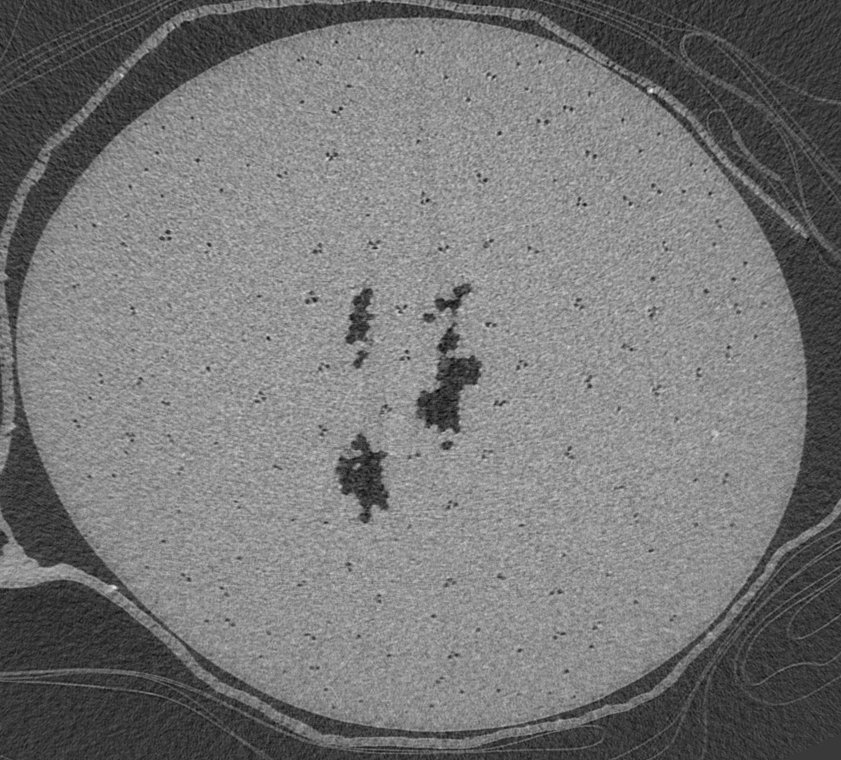

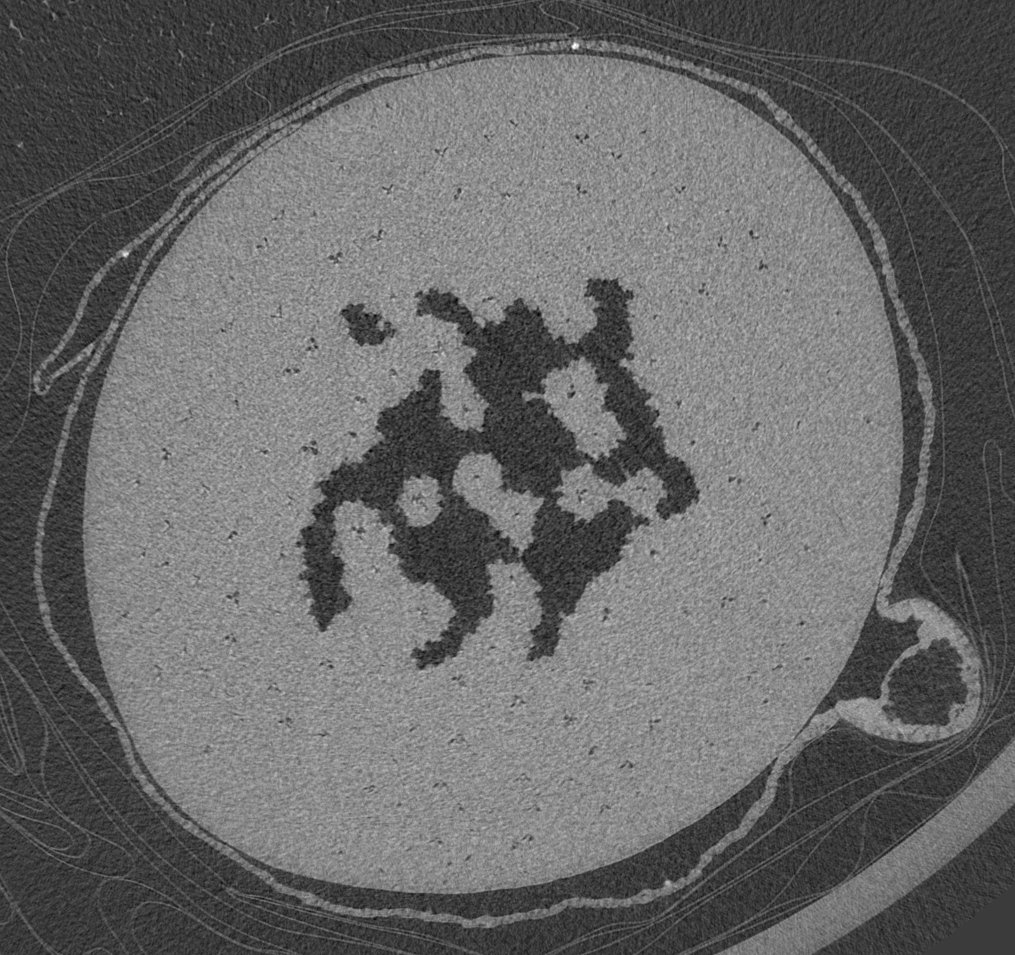


B73, no water, Ψ_PD_ = -1.7MPa, Ψ_MD_ = -1.9

B73, no water, Ψ_PD_ = -1.3MPa, Ψ_MD_ = -1.6


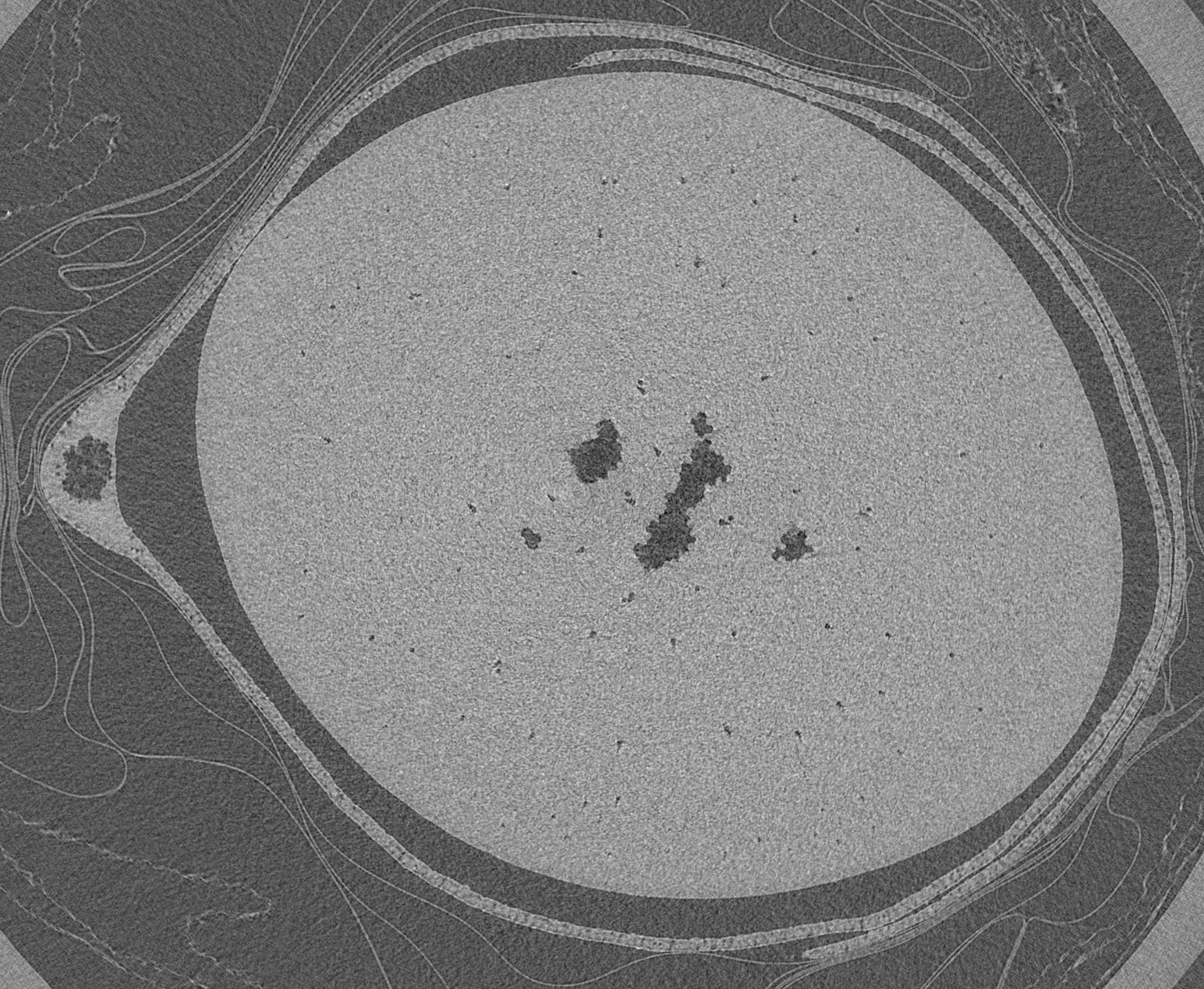

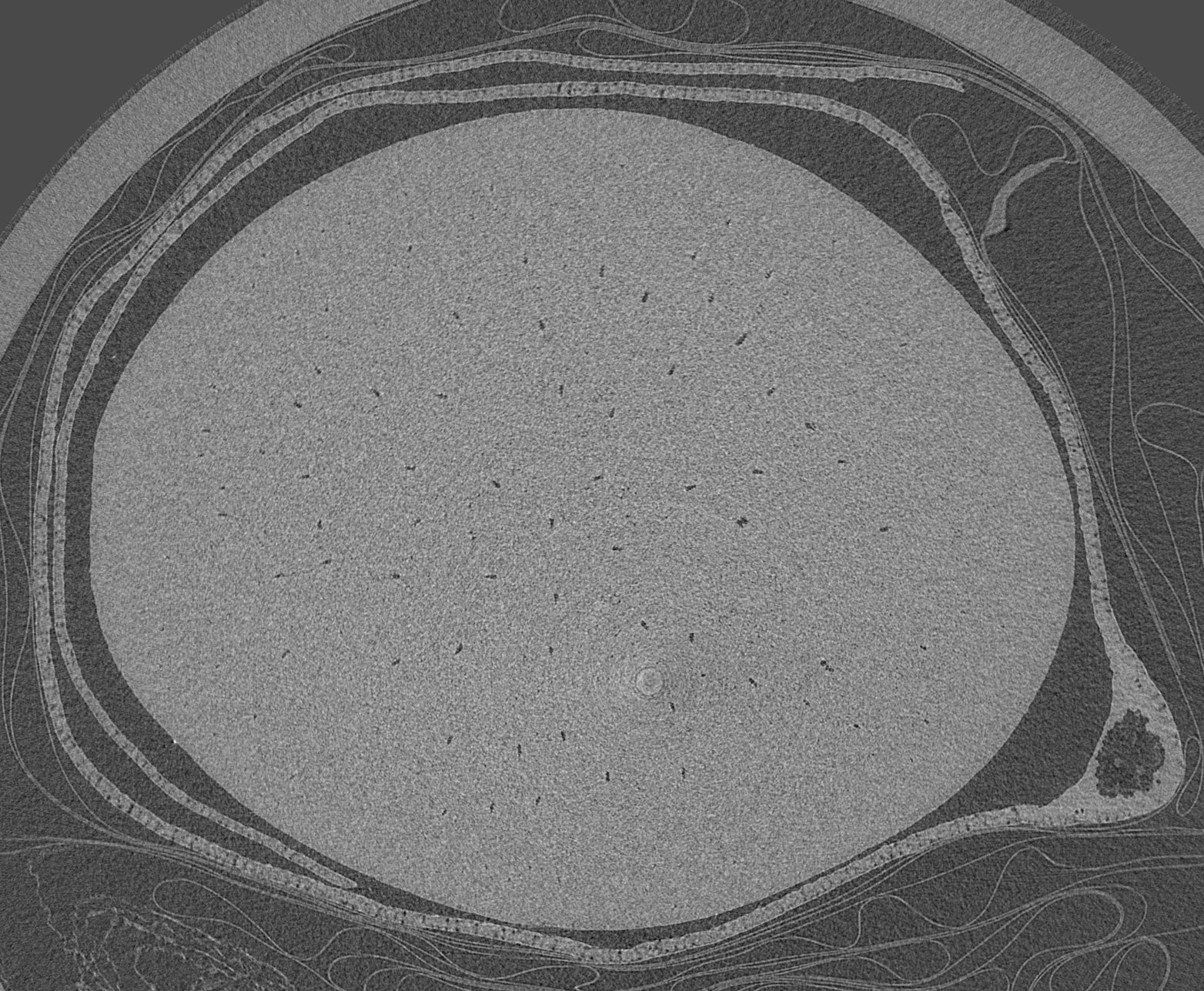


CML103, no water, Ψ_PD_ = -1.5MPa, Ψ_MD_ = -1.3

CML103, no water, Ψ_PD_ = -1.2MPa, Ψ_MD_ = -1.3
