# Supplementary material for: Embolized Stems Recover Overnight in Zea mays: The Role of Soil Water, Root Pressure, and Nighttime Transpiration
Source: Front Plant Sci. 2017 Apr 28;8:662. doi: 10.3389/fpls.2017.00662 (PMC5408072; doi:10.3389/fpls.2017.00662)
Supplement: Supplementary file 2 [file Data_Sheet_2.DOCX]

Sean M. Gleason*, Dustin R. Wiggans, Clayton A. Bliss, Jason S. Young, Mitchell Cooper, Katie R. Willi, Louise H. Comas

* Corresponding author. Tel.: +1 9704927411

sean.gleason55@gmail.com

**Supplemental Figure S2.** Repeat micro-ct scans after ca 4 hours in the dark at room temperature. Red symbols denote embolized vessels. Panels on left represent scans done within 2 hours of collection, whereas panels on right represent scans done within 5 hours of collection.


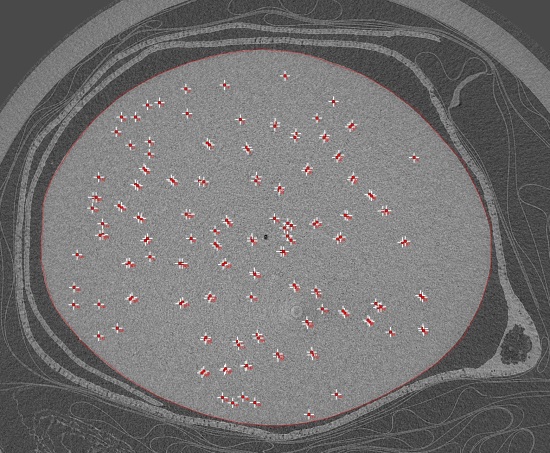

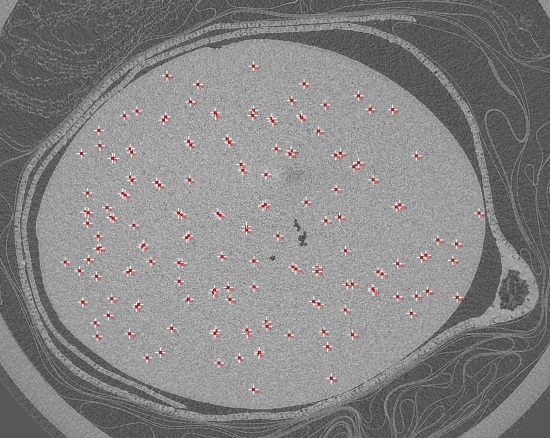


CML103, no water, Ψ_PD_ = -1.5MPa, scanned within 5h Dysfunctional vessel count = 164 of 603

CML103, no water, Ψ_PD_ = -1.5MPa, scanned within 2h Dysfunctional vessel count = 152 of 603


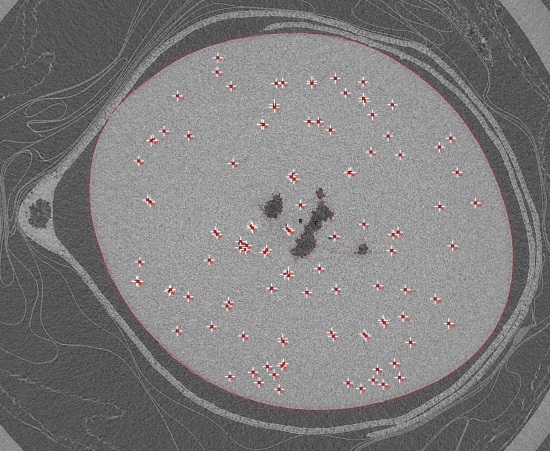

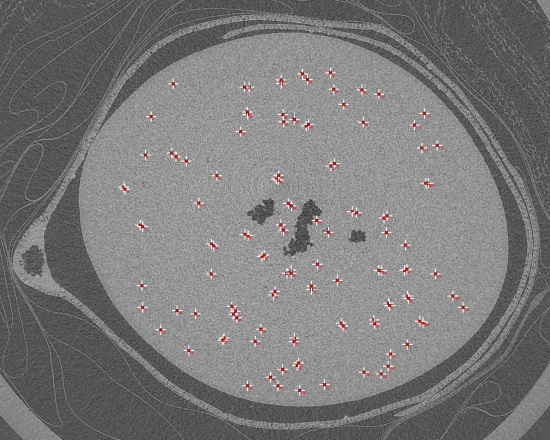


CML103, no water, Ψ_PD_ = -1.2MPa, scanned within 5h Dysfunctional vessel count = 117 of 567

CML103, no water, Ψ_PD_ = -1.2MPa, scanned within 2h Dysfunctional vessel count = 110 of 567
